# Exploring the potential targets and possible mechanisms of Shenqi Fuzheng Injection in the treatment of cancer-associated muscle wasting based on network pharmacology

**DOI:** 10.3389/fgene.2026.1876819

**Published:** 2026-10-01

**Authors:** Chen Liu, Ling Cheng, Wenfeng Ye, Lina Zhang

**Affiliations:** 1 Department of Clinical Nutrition, The Sun Yat-sen University Cancer Center, Guangzhou, China; 2 State Key Laboratory of Oncology in South China, Guangdong Provincial Clinical Research Center for Cancer, Sun Yat-sen University Cancer Center, Guangzhou, China; 3 Department of Medical Oncology, Guangzhou Institute of Cancer Research, The Affiliated Cancer Hospital, Guangzhou Medical University, Guangzhou, China; 4 Department of Clinical Nutrition, The Sixth Affiliated Hospital, Sun Yat-sen University, Guangzhou, China; 5 Guangzhou(Huangpu) Zhongliu Biomedical Innovation, Guangzhou, China

**Keywords:** cancer-associated muscle wasting, molecular docking, myoblasts, network pharmacology, Shenqi Fuzheng Injection

## Abstract

Cancer-associated muscle wasting (CAMW) is a debilitating condition observed in cancer patients, characterized by progressive skeletal muscle loss driven by tumor-secreted inflammatory cytokines, metabolic reprogramming, and enhanced apoptotic signaling, severely affecting the quality of life of patients. Shenqi Fuzheng Injection (SFI) is a traditional Chinese medicine preparation made from Codonopsis pilosula (Dangshen) and Astragalus membranaceus (Huangqi) in specific proportions, which has the effects of replenishing qi, strengthening the body, alleviating the side effects of radiotherapy and chemotherapy, relieving fatigue, and enhancing immune function. This study aims to explore the potential mechanisms of SFI in cancer-associated muscle wasting—a clinically significant form of sarcopenia frequently observed in cancer patients—through network pharmacology and molecular docking techniques. The key components in Shenqi Fuzheng Injection were found to have strong binding affinity with Caspase-3, Caspase-8, TNF, and TP53. Cell experiments further verified the effects of Shenqi Fuzheng Injection in alleviating mitochondrial damage in myoblasts and reducing apoptosis. Comprehensive analysis indicates that Shenqi Fuzheng Injection may target Caspase-3 and Caspase-8 to reduce apoptosis, thereby achieving therapeutic effects on cancer-associated muscle wasting. This study is exploratory and hypothesis-generating in nature; further *in vivo* validation is required to confirm these computational predictions.

## 1 Background

The term “Sarcopenia” (Sp) originates from Greek, where “sar” means muscle and “penia” means loss. In 1989, Irwin Rosenberg from Tufts University in the United States first proposed this concept (Fielding et al., 2011), initially defining it as age-related loss of skeletal muscle mass and strength. Primary sarcopenia is associated with aging, while secondary sarcopenia is caused by chronic diseases or loss of mobility. The present study specifically focuses on cancer-associated muscle wasting (CAMW), a clinically significant manifestation of secondary sarcopenia driven by tumor-related metabolic and inflammatory alterations, which is distinct from classical age-related primary sarcopenia in its etiology yet shares key molecular pathways including elevated TNF-α signaling, apoptotic cascades, and mitochondrial dysfunction. In clinical oncology settings, sarcopenia frequently co-occurs with cancer, manifesting as cancer-associated muscle wasting driven by overlapping mechanisms including elevated inflammatory cytokines (e.g., TNF-α), increased apoptotic signaling, and mitochondrial dysfunction (Biolo et al., 2014). This cancer-associated form shares key molecular pathways with age-related sarcopenia, justifying the investigation of shared therapeutic targets. The definition of sarcopenia includes reduced muscle mass, decreased muscle strength, and/or impaired physical function. A systematic review and meta-analysis found that sarcopenia is significantly associated with adult mortality, and this association is independent of population, definition, follow-up period, and risk of bias (Xu et al., 2021). This underscores the importance of early screening, diagnosis, and timely intervention and treatment in all populations. Research into the pathogenesis of sarcopenia is crucial for developing treatment plans and selecting therapeutic drugs. As a global disease, sarcopenia is influenced by multiple factors including age, disease, physical activity, nutritional status, hormone levels, and genetic factors. The discovery and development of effective drugs for sarcopenia hold significant value for clinical research.

Sarcopenia is an emerging disease, and there is no clear corresponding disease name in traditional Chinese medicine (TCM) literature. TCM categorizes the clinical manifestations of emaciated muscles and weak limbs as “Wei Syndrome,” believing that spleen deficiency leads to muscle weakness, and liver blood deficiency results in insufficient nourishment of tendons and vessels. According to the TCM theory that “the spleen governs the muscles,” there is a physiological and pathological correlation between the spleen and muscles, making the spleen the key organ in the differentiation of sarcopenia. The spleen and stomach are the source of qi and blood; when the spleen is strong, qi and blood flourish, and when the spleen is diseased, the limbs become weak and useless. The theory of treating from the spleen has good feasibility in TCM treatment of sarcopenia.

In Shenqi Fuzheng Injection (SFI), the monarch drug Astragalus (Huangqi) can tonify qi, elevate yang, strengthen the defensive qi, and promote diuresis and detoxification. The minister drug Codonopsis (Dangshen) can tonify the middle, benefit qi, generate fluids, nourish blood, and support the body’s resistance to eliminate pathogens. Both drugs are used in combination, both belonging to the spleen and lung meridians, and together they enhance the qi of the spleen and lungs. Codonopsis primarily tonifies the middle, while Astragalus tonifies qi and elevates yang. When used together, sufficient qi leads to the generation of blood, achieving dual tonification of qi and blood. The combination of these two drugs is dynamic yet not stagnant. Astragalus strengthens the defensive qi and resists external pathogens; Codonopsis tonifies the lung and spleen, supporting the body’s vital energy, forming a “dual internal and external” defense system. This drug has the effects of tonifying qi, supporting the body’s resistance, alleviating the side effects of radiotherapy and chemotherapy, relieving tumor fatigue, and enhancing the body’s immunity.

Network pharmacology constructs complex interaction networks of “drug-target-disease” using relevant software and algorithms to reveal the relationships between drug components, targets, and diseases, thereby predicting the intervention effects of drug components on diseases and exploring the multi-component, multi-target mechanisms of TCM (Hopkins, 2008; Jing et al., 2019). Based on network pharmacology, this study aims to explore the feasibility of Shenqi Fuzheng Injection in treating sarcopenia and its potential therapeutic targets, providing a theoretical basis for subsequent research and clinical application.

## 2 Materials and methods

### 2.1 Network pharmacology

The main chemical components of Codonopsis pilosula and Astragalus membranaceus were identified through databases such as the Traditional Chinese Medicine Systems Pharmacology Database and Analysis Platform (TCMSP (Ru et al., 2014) http://lsp.nwu.edu.cn/index.php) and the Taiwan Traditional Chinese Medicine Database (http://tcm.cmu.edu.tw/zh-tw/), as well as literature searches. Key compounds with high activity in Astragalus and Codonopsis were screened based on the criteria of oral bioavailability (OB) ≥30% and drug-likeness (DL) ≥0.18. It should be noted that these pharmacokinetic parameters (OB and DL) were originally designed for orally administered drugs and represent a methodological limitation when applied to an intravenous preparation such as SFI, as intravenous administration bypasses gastrointestinal absorption and first-pass metabolism. These criteria were applied as a conventional approach consistent with available TCM databases (TCMSP/TCMID (Xue et al., 2013) (http://www.megabionet.org/tcmid/)) and to maintain comparability with published network pharmacology studies on TCM preparations. Future studies should employ LC-MS/MS-based compound profiling of SFI to directly identify circulating active components.

Disease targets were retrieved from the CTD (CTD, 2021) (http://ctdbase.com/) database using the keywords ‘sarcopenia’, ‘muscle atrophy’, and ‘muscle wasting’, limited to *Homo sapiens*, using the curated gene-disease association subset to ensure data quality. “muscle atrophy” and “muscle wasting” keywords appropriately capture CAMW-related genes; noted future supplementation with “cancer cachexia”. Gene names were unified through the Uniprot (Wang et al., 2021) (https://www.uniprot.org/). The String (Szklarczyk et al., 2021) (https://cn.string-db.org/) database was used to construct protein-protein interaction networks, with a minimum required interaction score of 0.700 (high confidence threshold), limited to *Homo sapiens*. Network topology analysis was performed using Python’s NetworkX (Hagberg et al., 2008) package. KEGG (Kanehisa and Goto, 2000) (Kyoto Encyclopedia of Genes and Genomes) and GO database (Ashburner, 2021) (geneontology.org) enrichment analyses were conducted using a *p* < 0.05 threshold for the TOP20 results.

### 2.2 Molecular docking

Among the 41 candidate compounds, quercetin, kaempferol and luteolin were selected for molecular docking because they showed the highest degree of connectivity with the top core targets (CASP3, CASP8, TNF, MAPK3 and TP53) in the component-target network (Figure 1D). Structural files of the selected compounds and target proteins were obtained from the PDB and PubChem databases. Molecular docking was performed using CB-Dock2. CB-Dock2 employs a cavity detection method based on protein surface curvature (CurPocket) to guide molecular docking with AutoDock Vina. During the quality control phase, CB-Dock2 first checks the submitted protein structure files, adds missing side chain atoms and hydrogen atoms, detects missing protein residues, and removes co-crystallized water and other hetero groups. Subsequently, docking is performed based on the FitDock pipeline. A Vina score ≤ −7.0 kcal/mol was considered to indicate strong binding ability. Docking scores report predicted binding affinity, not pharmacological efficacy; functional validation was therefore assessed using the whole SFI preparation in cell experiments.

**FIGURE 1:**
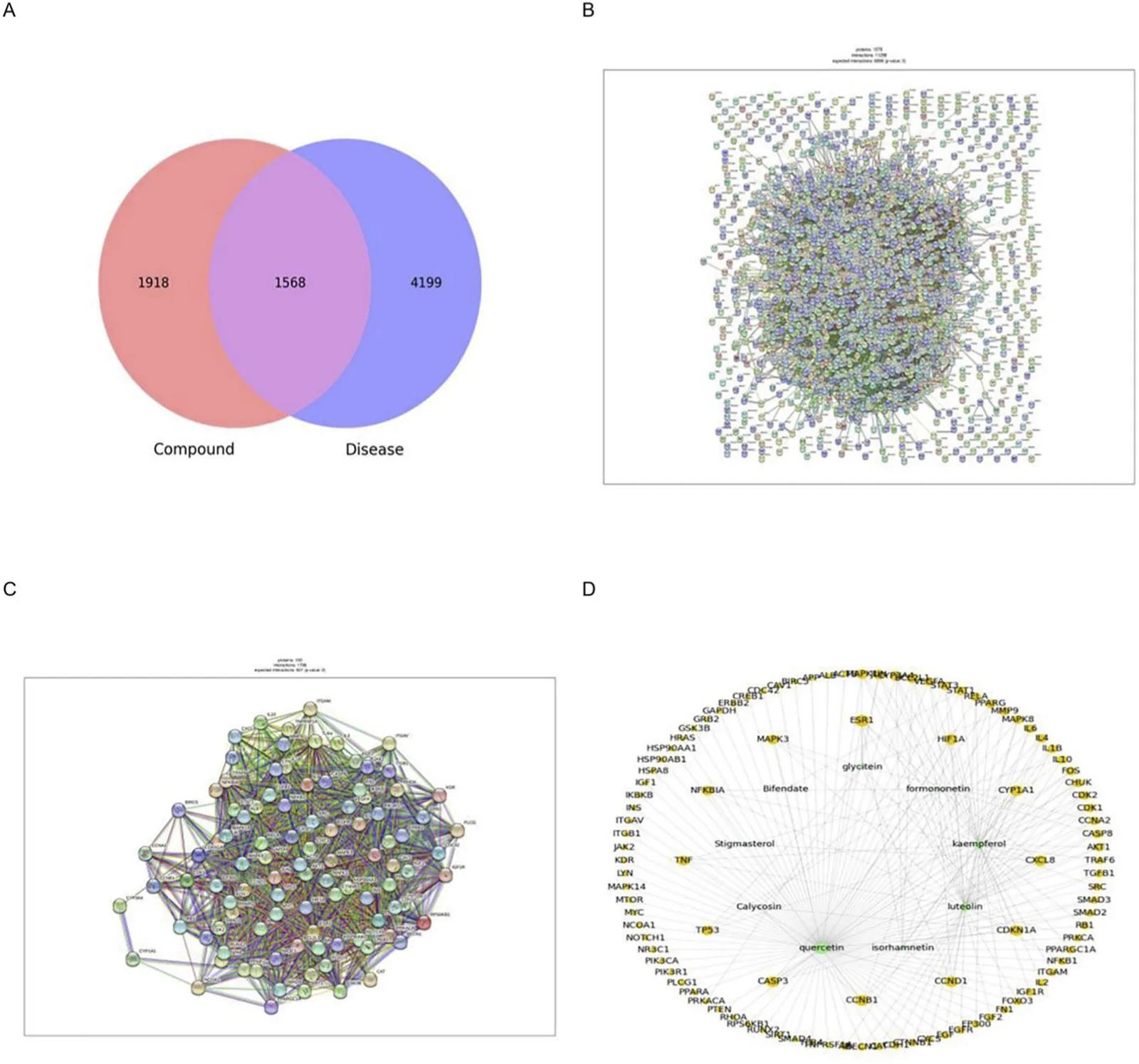
Construction and analysis of the component-target network of Shenqi Fuzheng Injection (SFI) in sarcopenia. **(A)** Venn diagram showing the intersection of SFI compound targets (n = 3,486) and sarcopenia-related genes (n = 5,767); 1,568 shared targets were retained for downstream analysis. **(B)** Protein-protein interaction (PPI) network of Astragalus- and Codonopsis-related targets (high-confidence score ≥0.700). **(C)** PPI network of the core targets shared between SFI and sarcopenia. **(D)** Component-core target network linking the representative SFI compounds (yellow diamonds) to core targets (coloured circles). Network visualisation was performed with Cytoscape/STRING; node size and colour reflect degree centrality.

### 2.3 Cell experiments

#### 2.3.1 Effect of Shenqi Fuzheng Injection on myoblast proliferation

FaDu cells are a human pharyngeal squamous carcinoma cell line, catalog number CL-0083; C2C12 cells are a mouse myoblast cell line, catalog number CL-0044. Both cell lines were purchased from Wuhan Pricella Life Science Technology Co., Ltd. Both cell lines were maintained in DMEM supplemented with 10% fetal bovine serum (FBS), 100 U/mL penicillin, and 100 μg/mL streptomycin at 37 °C in a humidified atmosphere of 5% CO_2_. *Mycoplasma* testing was performed prior to experiments using a PCR-based *mycoplasma* detection kit.

SFI concentrations (5, 10 and 20 mg/mL) were selected as follows. A standard clinical infusion of SFI is 250 mL, with a specification of 1 mL equivalent to 1 g crude drug, delivering a total crude-drug dose of 250 g per infusion. For a 70-kg adult with an estimated total body water volume of ∼42 L (60% of body weight), the nominal peak systemic concentration is approximately 250 g/42 L ≈ 6 mg/mL. Because plasma protein binding, tissue partitioning and *in vitro* dilution modify effective exposure, we bracketed this estimate with 5, 10 and 20 mg/mL. CCK-8 pre-screening confirmed these concentrations were non-cytotoxic in C2C12 myoblasts (relative viability ∼95–105% of control; all p > 0.05 vs. control). Concentrations >20 mg/mL were avoided to prevent osmotic or vehicle artefacts.

#### 2.3.2 Effect of Shenqi Fuzheng Injection on myoblast apoptosis

The co-culture system was evaluated at 6 h, 12 h and 24 h to determine the optimal apoptosis-induction time point. Apoptosis peaked at 12 h and therefore this time point was chosen for subsequent SFI intervention experiments. Later time points (e.g., 48 h) were not assessed by Annexin V/PI in this assay because prolonged co-culture increases secondary necrosis, cell detachment and loss of viability, which confound the interpretation of apoptotic fractions. The 6–24 h window therefore captures the acute paracrine injury phase relevant to cancer-associated muscle wasting.

#### 2.3.3 Detection of mitochondrial membrane potential in myoblasts treated with Shenqi Fuzheng Injection

JC-1 staining was used to assess mitochondrial membrane potential. Cells were washed with PBS, incubated with JC-1 working solution at 37 °C for 20 min, washed twice with JC-1 staining buffer, and observed under fluorescence microscopy.

#### 2.3.4 Western blot to detect the expression of caspase 3, caspase 8 protein

Three groups were assigned: C2C12 (control), C2C12 + FaDu (model), and C2C12 + FaDu + SFI (treatment). Total protein was extracted using RIPA lysis buffer with protease inhibitors and quantified by BCA assay. 20 μg protein per sample was separated by SDS-PAGE and transferred to PVDF membranes. Membranes were blocked with 5% non-fat milk in TBST for 1 h at room temperature, then incubated with primary antibodies overnight at 4 °C: anti-Caspase-3 (1:2000, Cell Signaling Technology, ab184787), anti-Caspase-8 (1:1000, Cell Signaling Technology, ab25901), GAPDH Loading Control Antibody (1:10,000, ThermoFish, MA5-1573). HRP-conjugated secondary antibodies (1:5000) were incubated for 1 h at room temperature. Protein bands were visualized using ECL substrate and imaged.

### 2.4 Statistical analysis

All quantitative data are presented as mean ± standard deviation (SD). Comparisons between two groups were performed using Student’s t-test. Multiple group comparisons were analyzed by one-way ANOVA followed by Tukey’s post-hoc test. All statistical analyses were performed using GraphPad Prism 9.0 (GraphPad Software, San Diego, CA). A *p* value <0.05 was considered statistically significant.

## 3 Results

### 3.1 Screening results of chemical components in Shenqi Fuzheng Injection

Using OB ≥ 30% and DL ≥ 0.18 as screening criteria, 41 potential active compounds were obtained (20 from Astragalus, 21 from Codonopsis). Representative compounds including Quercetin, Kaempferol, and Luteolin are summarized in Table 1.

**TABLE 1:** Potential active compounds of astragalus and codonopsis.

ID	Compound name	OB (%)	DL
MOL001006	Poriferasta-7,22E-dien-3beta-ol	42.98	0.76
MOL002140	Perlolyrine	65.95	0.27
MOL002879	Diop	43.59	0.39
MOL003036	ZINC03978781	43.83	0.76
MOL000449	Stigmasterol	43.83	0.76
MOL003896	7-Methoxy-2-methyl isoflavone	42.56	0.2
MOL004355	Spinasterol	42.98	0.76
MOL005321	Frutinone A	65.9	0.34
MOL000006	Luteolin	36.16	0.25
MOL006774	Stigmast-7-enol	37.42	0.75
MOL007059	3-beta-Hydroxymethyllenetan shiquinone	32.16	0.41
MOL007514	Methyl icosa-11,14-dienoate	39.67	0.23
MOL008397	Daturilin	50.37	0.77
MOL004492	Chrysanthemaxanthin	38.72	0.58
MOL006554	Taraxerol	38.4	0.77
MOL008400	Glycitein	50.48	0.24
MOL008407	(8S,9S,10R,13R,14S,17R) -17-[(E,2R,5S)-5-ethyl-6-methylhept-3-en-2-yl]-10,13-dimethyl-1,2,4,7,8,9,11,12,14, 15,16,17-dodecahydrocyclopenta [a]phenanthren-3-one	45.4	0.76
MOL008411	11-Hydroxyrankinidine	40	0.66
MOL008391	5alpha-Stigmastan-3,6-dione	33.12	0.79
MOL008393	7- (β-Xylosyl)thujopsene 8- 8-[7-(beta-Xylosyl) 9-cephalomannine_qt]	38.33	0.29
MOL008406	Spinoside A	39.97	0.4
MOL000211	Mairin	55.38	0.78
MOL000239	Jaranol	50.83	0.29
MOL000296	Hederagenin	36.91	0.75
MOL000033	(3S,8S,9S,10R,13R,14S,17R) -10,13-dimethyl-17-[(2R,5S) -5-propan-2-yloctan-2-yl] -2,3,4,7,8,9,11,12,14,15,16,17 -dodecahydro-1H-cyclopenta [a] phenanthren-3-ol	36.2	0.78
MOL000354	Isorhamnetin	49.6	0.31
MOL000371	3,9-di-O-methylnissolin	53.74	0.48
MOL000374	5′-hydroxyiso-muronulatol-2′, 5′-di-O-glucoside	41.72	0.69
MOL000378	7-O-methylisomucronulatol	74.69	0.3
MOL000379	9,10-dimethoxypterocarpan -3-O-β-D-glucoside	36.74	0.92
MOL000380	(6aR,11aR)-9,10-dimethoxy-6a,	64.26	0.42
	11a-dihydro-6H-benzofurano [3,2-c]chromen-3-ol		
MOL000387	Bifendate	31.1	0.67
MOL000392	Formononetin	69.67	0.21
MOL000398	Isoflavanone	109.99	0.3
MOL000417	Calycosin	47.75	0.24
MOL000422	Kaempferol	41.88	​
MOL000433	FA	68.96	0.71
MOL000438	(3R)-3-(2-hydroxy-3, 4-dimethoxyphenyl)chroman-7-ol	67.67	0.26
MOL000439	Isomucronulatol-7,2′-di-O-glucosiole	49.28	0.62
MOL000442	1,7-Dihydroxy-3, 9-dimethoxy pterocarpene	39.05	0.48
MOL000098	Quercetin	46.43	0.28

### 3.2 Component-target network construction and analysis

A total of 3,486 TCM component targets and 5,767 sarcopenia-related genes (from CTD using keywords ‘sarcopenia’, ‘muscle atrophy’, ‘muscle wasting’) were identified, yielding 1,568 common targets. PPI network analysis identified the top 20 core targets including TP53, AKT1, TNF, CASP3, CASP8, MAPK3, STAT3, IL6, and others (Figure 1).

### 3.3 Functional enrichment analysis of core target genes

GO enrichment analysis revealed 4,136 biological process terms, 283 cellular component terms, and 417 molecular function terms (*p* < 0.05, TOP20). These results are interpreted in the context of sarcopenia pathobiology: (1) Biological processes—'response to peptide’ and ‘epithelial cell proliferation’ reflect growth factor-mediated muscle cell signaling (e.g., IGF-1, EGF pathways); ‘myeloid cell differentiation’ is connected to the chronic inflammatory microenvironment central to sarcopenia; ‘wound healing’ relates to impaired muscle regeneration capacity. (2) Molecular functions—'ubiquitin protein ligase binding’ and ‘DNA-binding transcription factor binding’ are directly relevant to the ubiquitin-proteasome pathway (UPP), the primary protein degradation pathway in sarcopenic muscle, involving E3 ligases Atrogin-1 and MuRF1. (3) Cellular components—'membrane raft’ and ‘secretory granule lumen’ relate to cytokine secretion and TNF-α/IL-6-mediated inflammatory signaling driving muscle catabolism (Figure 2).

**FIGURE 2:**
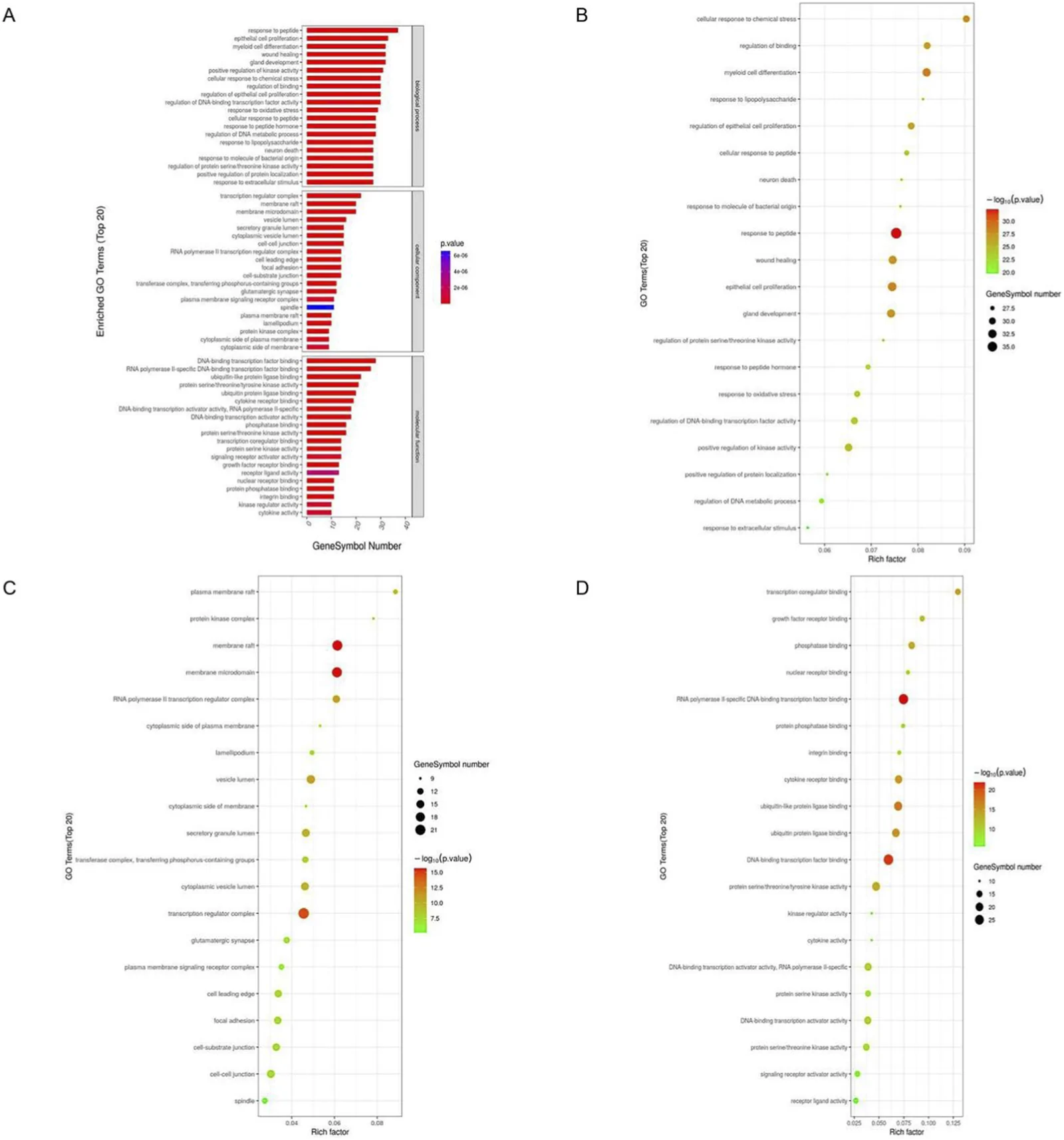
Gene Ontology (GO) enrichment analysis of the 1,568 SFI-sarcopenia shared targets (p < 0.05, TOP 20). **(A)** Summary bar plot of enriched GO terms across biological process (BP), cellular component (CC) and molecular function (MF). **(B)** Bubble plot of biological process terms; the x-axis indicates rich factor and bubble size reflects the number of genes. **(C)** Bubble plot of cellular component terms. **(D)** Bubble plot of molecular function terms. Colour scale represents −log10 (p value).

### 3.4 Signal pathway analysis of core targets

KEGG enrichment analysis identified 122 pathway entries (*p* < 0.05, TOP20). While cancer pathways dominated the enrichment results—a recognized limitation of TCM network pharmacology studies due to the substantial overlap between TCM targets and oncological research—several pathways are directly relevant to sarcopenia pathophysiology: (1) MAPK signaling pathway: regulates muscle cell inflammatory responses, protein synthesis/degradation balance, and cellular senescence; (2) Apoptosis pathway: Caspase-3/8-mediated muscle cell death is a core mechanism in sarcopenia; (3) PI3K-Akt signaling: central regulator of protein synthesis (mTOR) and muscle hypertrophy/atrophy balance; (4) TNF signaling: mediates chronic low-grade inflammation driving muscle proteolysis in sarcopenia. We caution against direct over-interpretation of cancer-specific pathway involvement; the enrichment reflects the shared molecular machinery between cancer and sarcopenia. The background gene set for enrichment analysis comprised the 1,568 common targets identified from the intersection of SFI component targets and sarcopenia disease targets (Figure 3).

**FIGURE 3:**
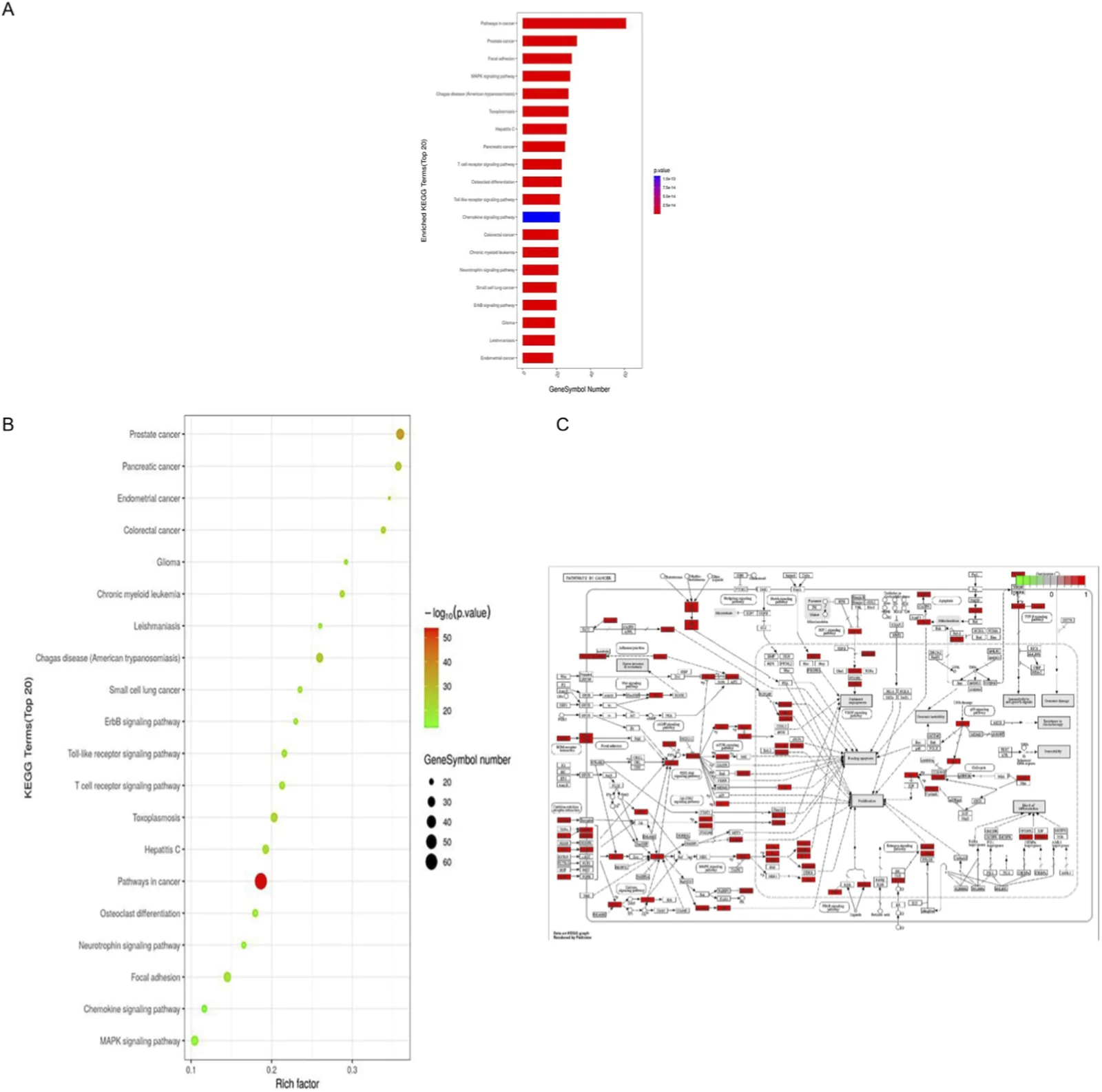
Kyoto Encyclopedia of Genes and Genomes (KEGG) pathway enrichment analysis of SFI-sarcopenia shared targets (p < 0.05, TOP 20). **(A)** Bar plot of enriched KEGG pathways ranked by gene count. Pathways directly relevant to muscle wasting (MAPK, apoptosis, TNF, PI3K-Akt) are highlighted. **(B)** Bubble plot showing rich factor, gene number and −log10 (p value). **(C)** KEGG pathway diagram illustrating the position of core targets within the pathways in cancer/apoptosis network. Note that cancer-related pathway enrichment reflects the shared molecular machinery between tumour biology and muscle wasting rather than a primary cancer mechanism.

### 3.5 Molecular docking

Molecular docking confirmed strong binding affinity (Vina score ≤ −7.0 kcal/mol) of Quercetin, Luteolin, and Kaempferol with Caspase-3, Caspase-8, TNF, MAPK3, and TP53 (Table 2; Figure 4).

**TABLE 2:** Molecular docking results of key components with core targets.

Compounds	Proteins	ID	Cavities volume	Center x	Center y	Center z	Size x	Size y	Size z	Vina score
Quercetin	Caspase-3	1	392	11	28.6	45	21	21	21	−6
​	​	2	284	37	34.7	32	21	21	21	−7
​	​	3	194	21	6.99	35	21	21	21	−7
​	​	4	130	42	24.2	26	21	21	21	−7
​	​	5	110	27	30.6	48	21	21	21	−6
​	Caspase-8	1	1146	−9	15.08	−36.2	27	21	34	−8.1
​	​	2	233	11	2.055	−37.5	21	21	21	−7.7
​	​	3	207	−27	14.97	−32.8	21	21	21	−7.3
​	​	4	174	−23	29.77	−47.5	21	21	21	−6.9
​	​	5	161	−4	1.567	−48	21	21	21	−6.9
​	MAPK3	1	923	40	4	−11	21	21	21	−6.9
​	​	2	916	47	−12	−4	21	29	21	−7.6
​	​	3	755	55	1	−1	21	21	29	−8
​	​	4	692	34	−11	−20	21	21	21	−8.5
​	​	5	404	56	−17	−18	21	21	21	−6
​	TNF	1	1327	−32	−93	54	21	21	21	−7.4
​	​	2	808	−34	−76	60	21	29	21	−9.2
​	​	3	651	−18	−83	63	21	21	32	−6.8
​	​	4	503	−15	−51	55	21	21	21	−8.2
​	​	5	424	−37	−88	74	21	21	21	−9.7
​	TP53	1	3691	28	−2	29	28	21	21	−7.7
​	​	2	882	11	−16	20	21	21	21	−7.5
​	​	3	730	14	−32	8	21	21	21	−7.4
​	​	4	581	24	−26	5	21	21	21	−7.6
​	​	5	580	25	−24	20	21	21	21	−6.7
Luteolin	Caspase-3	1	279	22	33	62	21	21	21	−7.1
​	​	2	235	12	11	50	21	21	21	−5.9
​	​	3	191	18	30	43	21	21	21	−7.1
​	​	4	169	27	12	50	21	21	21	−6.1
​	​	5	144	34	5	67	21	21	21	−6.6
​	Caspase-8	1	1146	−9	15	−36	27	21	34	−7.9
​	​	2	233	11	2	−38	21	21	21	−7.7
​	​	3	207	−27	15	−33	21	21	21	−7.2
​	​	4	174	−23	30	−48	21	21	21	−6.7
​	​	5	161	−4	2	−48	21	21	21	−7.2
​	TNF	1	1327	−32	−93	54	21	21	21	−7.4
​	​	2	808	−34	−76	60	21	21	21	−8.9
​	​	3	651	−18	−83	63	21	21	32	−7.4
​	​	4	503	−15	−51	55	21	21	21	−8.5
​	​	5	424	−37	−88	74	21	21	21	−9.3
​	TP53	1	3691	28	−2	29	28	21	21	−8.1
​	​	2	882	11	−16	20	21	21	21	−7.7
​	​	3	730	14	−32	8	21	21	32	−7.8
​	​	4	581	24	−26	5	21	21	21	−7.9
​	​	5	580	25	−24	20	21	21	21	−6.9
Kaempferol	Caspase-3	1	279	22	33	62	21	21	21	−6.3
​	​	2	235	12	11	50	21	21	21	−5.5
​	​	3	191	18	30	43	21	21	21	−7
​	​	4	169	27	12	50	21	21	21	−5.9
​	​	5	144	34	5	67	21	21	21	−6.5
​	Caspase-8	1	1146	−9	15	−36	27	21	34	−7.5
​	​	2	233	11	2	−38	21	21	21	−7.2
​	​	3	207	−27	15	−33	21	21	21	−7.3
​	​	4	174	−23	30	−48	21	21	21	−6.7
​	​	5	161	−4	2	−48	21	21	21	−6.8
​	MAPK3	1	923	40	4	−11	21	21	21	−6
​	​	2	916	47	−12	−4	21	29	21	−7.3
​	​	3	755	55	1	−1	21	21	29	−7.6
​	​	4	692	34	−11	−20	21	21	21	−7.5
​	​	5	404	56	−17	−18	21	21	21	−6
​	TNF	1	1327	−32	−93	54	21	21	21	−7.2
​	​	2	808	−34	−76	60	21	21	21	−8.5
​	​	3	651	−18	−83	63	21	21	32	−6.6
​	​	4	503	−15	−51	55	21	21	21	−8.2
​	​	5	424	−37	−88	74	21	21	21	−8.8
​	TP53	1	3691	28	−2	29	28	21	21	−7.8
​	​	2	882	11	−16	20	21	21	21	−7.2
​	​	3	730	14	−32	8	21	21	32	−7.4
​	​	4	581	24	−26	5	21	21	21	−8.4
​	​	5	580	25	−24	20	21	21	21	−6.8

**FIGURE 4:**
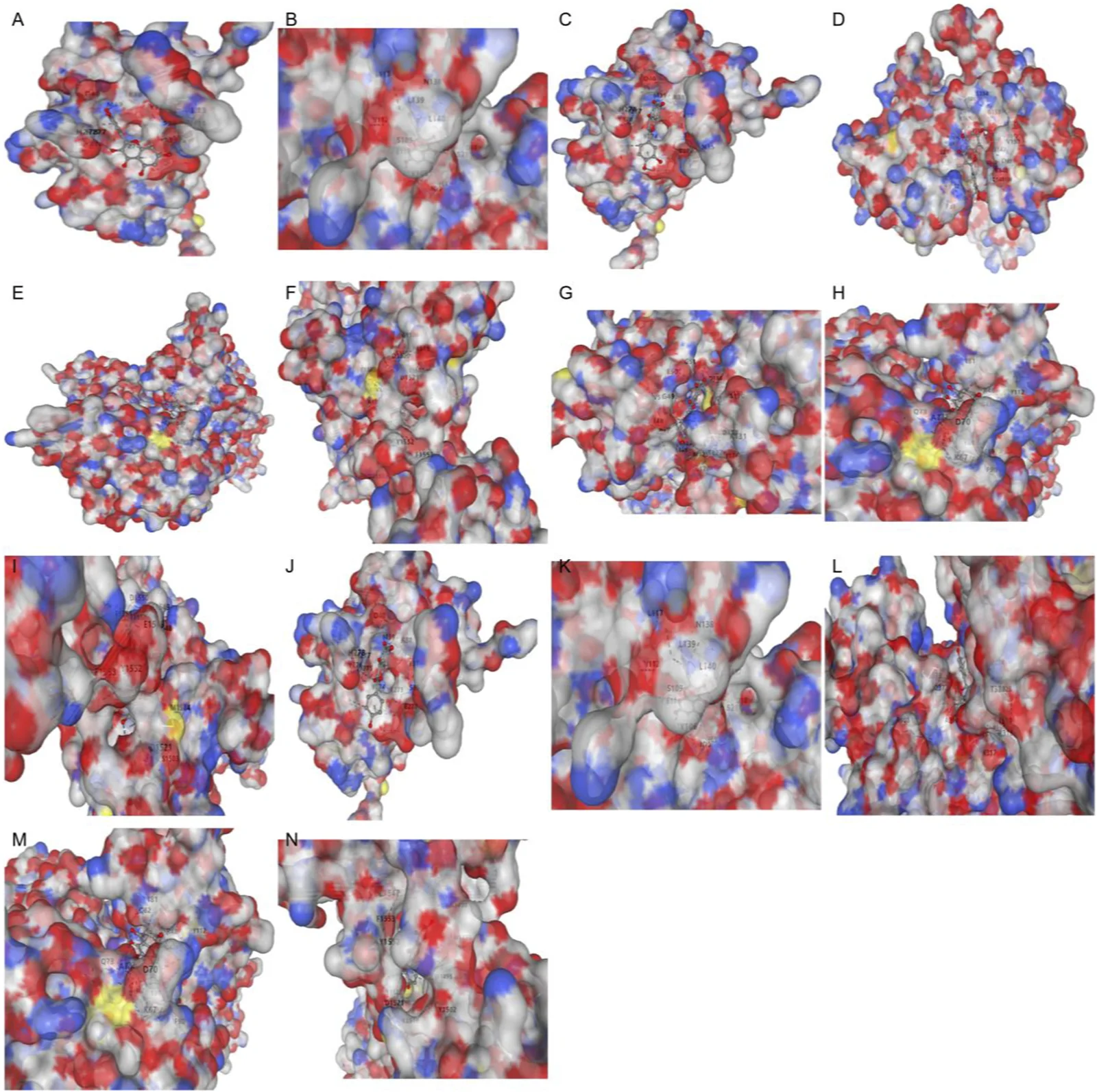
Representative molecular-docking poses of the three representative flavonoids with core sarcopenia targets (CB-Dock2/AutoDock Vina). Surface representations show the binding cavity and surrounding residues. **(A)** Quercetin–Caspase-3; **(B)** Quercetin–Caspase-8; **(C)** Luteolin–Caspase-3; **(D)** Luteolin–Caspase-8; **(E)** Luteolin–TNF; **(F)** Luteolin–TP53; **(G)** Quercetin–MAPK3; **(H)** Quercetin–TNF; **(I)** Quercetin–TP53; **(J)** Kaempferol–Caspase-3; **(K)** Kaempferol–Caspase-8; **(L)** Kaempferol–MAPK3; **(M)** Kaempferol–TNF; **(N)** Kaempferol–TP53. A Vina score ≤ −7.0 kcal/mol was considered strong binding affinity. Docking predicts binding pose, not functional activation or inhibition.

### 3.6 Cell experiments

Cell experiments showed the following quantitative findings (Figure 5). (1) C2C12 myoblast viability was not impaired by SFI at any tested concentration (0, 2.5, 5, 10 or 20 mg/mL) or time point (0, 6, 12, 24 or 48 h) in the CCK-8 assay (relative viability ∼95–105% of control; all p > 0.05 vs. control) (Figures 5A,B). (2) FaDu/C2C12 co-culture induced a time-dependent increase in Annexin V-positive apoptotic C2C12 cells, with peak apoptosis at 12 h and a decline by 24 h (model vs. C2C12-only control, p < 0.001) (Figure 5C). SFI at 5 and 10 mg/mL dose-dependently reduced this apoptotic response (both p < 0.01 vs. model). (3) JC-1 staining showed that co-culture reduced the red/green fluorescence ratio, indicating loss of mitochondrial membrane potential, whereas SFI restored red fluorescence and preserved mitochondrial membrane potential (Figure 5D). (4) Western blot quantification (relative to GAPDH) demonstrated that the model group markedly upregulated Caspase-3 and Caspase-8 protein expression (Caspase-3: 0.42 ± 0.02; Caspase-8: 0.42 ± 0.02) compared with the C2C12 control (Caspase-3: 0.06 ± 0.01; Caspase-8: 0.06 ± 0.01; both p < 0.001). SFI treatment significantly downregulated both proteins (Caspase-3: 0.21 ± 0.02; Caspase-8: 0.21 ± 0.02; both p < 0.001 vs. model) (Figure 5E).

**FIGURE 5:**
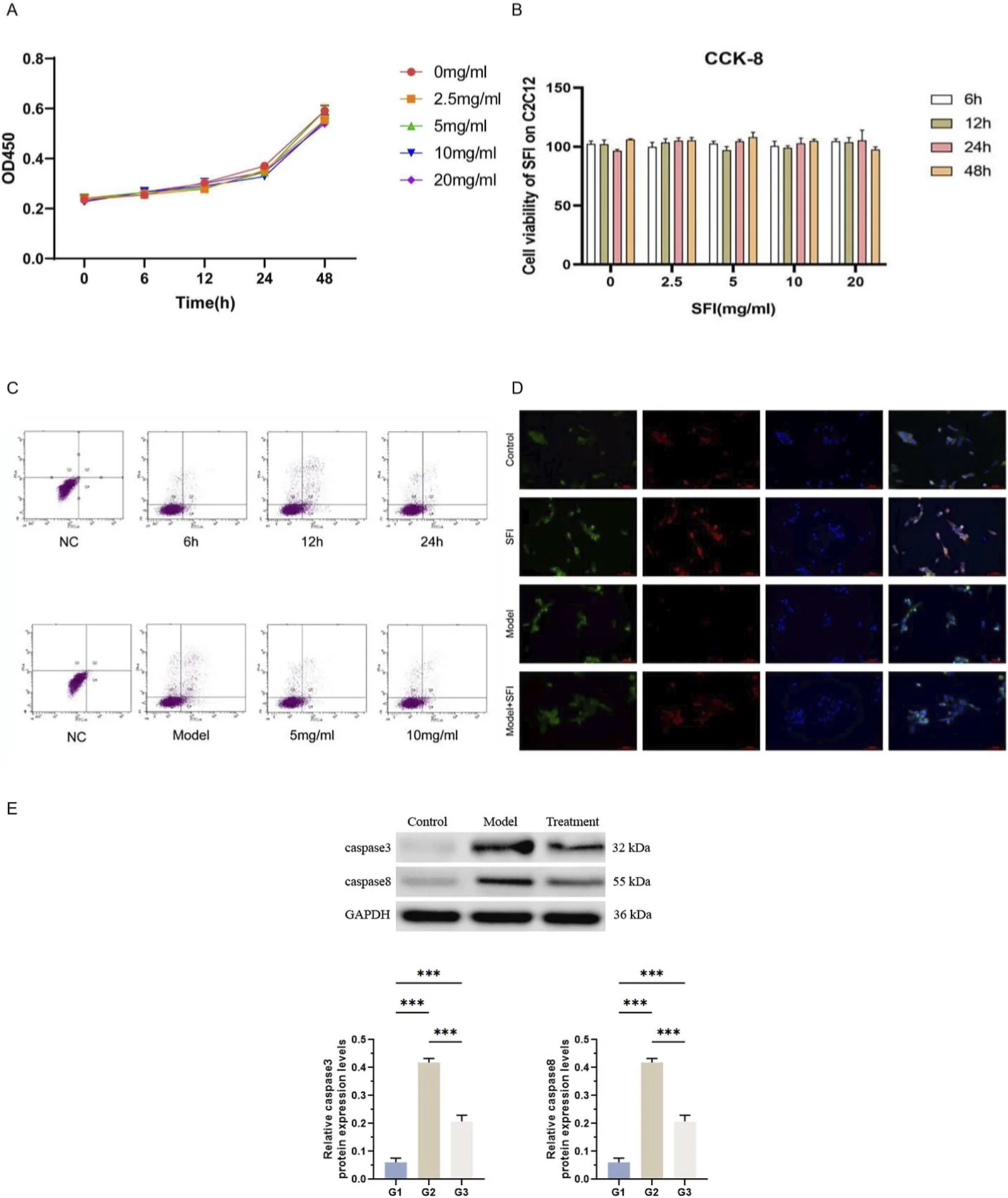
Shenqi Fuzheng Injection (SFI) protects C2C12 myoblasts from FaDu co-culture-induced mitochondrial injury and apoptosis. **(A)** CCK-8 time-course OD450 values for C2C12 cells exposed to 0–20 mg/mL SFI for 0, 6, 12, 24 and 48 h. **(B)** Relative C2C12 viability after SFI treatment at different concentrations and time points. Data are mean ± SD; all SFI groups vs. control, p > 0.05. **(C)** Annexin V/PI flow cytometry. Upper row: time-course of FaDu/C2C12 co-culture (NC, 6 h, 12 h, 24 h) showing peak apoptosis at 12 h. Lower row: effect of 0, 5 and 10 mg/mL SFI intervention at 12 h (NC, Model, 5 mg/mL, 10 mg/mL). **(D)** JC-1 mitochondrial membrane potential staining: green (JC-1 monomer), red (JC-1 aggregate), blue (nuclei) and merged images for Control, SFI alone, Model and Model + SFI groups. **(E)** Western blot and densitometric quantification of Caspase-3 and Caspase-8 protein expression relative to GAPDH. G1 = C2C12 control; G2 = C2C12 + FaDu model; G3 = C2C12+FaDu + SFI treatment. ***p < 0.001 vs. indicated group.

## 4 Discussion

Shenqi Fuzheng Injection is a pure traditional Chinese medicine preparation made from Codonopsis pilosula and Astragalus membranaceus in specific proportions through modern processing techniques. Its mechanism of action has been gradually clarified through extensive experimental research and clinical applications, mainly including regulating cell proliferation and apoptosis, improving cellular oxidative stress and mitochondrial dysfunction, enhancing immune function, increasing drug resistance, and improving chemotherapy sensitivity. However, as a compound preparation of traditional Chinese medicine, its components are more complex than single drugs, and the interaction of various pharmacological components in the treatment of sarcopenia and related signaling pathways are still insufficiently studied. In addition, the existing clinical data are mostly small-sample studies, lacking multicenter, large-sample clinical trial data to further verify its medicinal value and clinical significance.

From a traditional Chinese medicine (TCM) perspective, the SFI formula pairs Astragalus membranaceus (Huangqi) as the monarch herb to tonify qi and strengthen the exterior, with Codonopsis pilosula (Dangshen) as the minister herb to tonify the middle, benefit qi, generate fluid and nourish blood. This combination embodies the principle of “qi-blood dual tonification” and the TCM doctrine that “the spleen governs the muscles”. Modern pharmacological studies support this pairing: Astragalus-derived flavonoids and saponins exhibit anti-inflammatory, immunomodulatory and mitochondrial-protective effects, whereas Codonopsis polysaccharides and lobetyolin can improve gastrointestinal function, fatigue and nutritional status (Li et al., 2024; Zhu et al., 2019). Thus, the formula-level activity of SFI is not a simple sum of single-compound effects; rather, the Astragalus–Codonopsis combination likely targets multiple nodes (chronic inflammation, oxidative stress, mitochondrial dysfunction and apoptosis) that converge on cancer-associated muscle wasting (Cui et al., 2023).

Through network pharmacology research, it was found that Shenqi Fuzheng Injection is mainly enriched in tumor-related pathways in the treatment of muscle loss. After screening the main chemical components and therapeutic targets, it was found that its key components quercetin, kaempferol, and luteolin are closely related to caspase3, caspase8, MAPK3, TNF, and TP53. Quercetin, as a flavonol compound, has various pharmacological properties such as anti-cancer, anti-allergic, anti-inflammatory, and improving bone quality (Aghababaei and Hadidi, 2023). Kaempferol and luteolin are common flavonoid compounds with anti-inflammatory, antioxidant, and anti-tumor effects. Quercetin can enhance mitochondrial biogenesis, promote the formation of new mitochondria, and is crucial for improving muscle endurance and energy production. It activates AMPK and SIRT1, upregulating PGC-1α, which is a key regulator of mitochondrial biogenesis (Koshinaka et al., 2020; Eid et al., 2010; Tang, 2016). In high-fat diet mice, quercetin significantly reduces muscle inflammation and apoptosis by inhibiting the expression of atrophy factors such as Atrogin-1 and MuRF1, lowering skeletal muscle inflammatory cytokine levels and macrophage infiltration, thereby improving muscle quality (Le et al., 2014). Numerous studies have confirmed that quercetin can inhibit oxidative stress, inflammation, autophagy, matrix degradation, and apoptosis, promote muscle health, and induce migration and differentiation, improving muscle regeneration (Qu et al., 2021). Luteolin significantly enhances NRF2 activity in C2C12 cells, increasing the expression of NRF2 target genes (Böttcher et al., 2025). In addition, luteolin has anti-inflammatory and protein degradation inhibitory effects in high-fat diet-induced obese mice, can inhibit lipid infiltration in muscles, reduce p38 activity and the mRNA expression of inflammatory factors, affecting muscle protein degradation (Kim et al., 2023). Kaempferol, as a TAZ activator, exhibits myogenic effects in C2C12 cells (Kodaka et al., 2020).

This study used network pharmacology methods to preliminarily screen the active substances (quercetin, kaempferol, luteolin) and key targets (MAPK3, TP53, TNF, Caspase3, Caspase8) of Shenqi Fuzheng Injection in the treatment of sarcopenia, and analyzed its potential mechanisms through related signaling pathways, involving tumor, MAPK, apoptosis, and other pathways. Molecular docking verified the affinity of the compounds in Shenqi Fuzheng Injection with key targets. The MAPK signaling pathway plays a key role in cell proliferation, differentiation, migration, senescence, and apoptosis (Sun et al., 2015). IGF1 can alleviate age-related ICC/ICC-SC loss, improve gastric compliance, and increase food intake in klotho mice by activating the ERK1/2 signaling pathway, thereby alleviating frailty and sarcopenia (Nguyen et al., 2023). TP53, as a tumor suppressor gene, can regulate target gene expression, induce cell cycle arrest, apoptosis, senescence, DNA repair, or metabolic changes. Studies have shown that chronic activation of autophagy by muscle-specific overexpression of TRP53,INP2 can prevent sarcopenia and muscle function decline in mice, while acute re-expression of TRP53INP2 in aged mice can improve muscle atrophy, enhance mitochondrial function, and reduce ROS production. In humans, high levels of TP53,INP2 in muscles are associated with increased muscle strength and healthy aging (Sebastián et al., 2024). Tumor necrosis factor (TNF)-α is a pro-inflammatory cytokine involved in the pathogenesis of sarcopenia. Studies have found that plasma anti-TNF-α levels are positively correlated with TNF-α and significantly associated with sarcopenia (Lin et al., 2023). During tumor burden and aging, muscle mass decreases, leading to sarcopenia, which is associated with low-grade chronic inflammation. This inflammation promotes proteolysis of muscle fibers and inhibits their regeneration, thereby inducing sarcopenia. Studies have shown that by preventing TNF-α-induced proteolysis and inhibiting muscle fiber regeneration, sarcopenia related to age and rheumatoid arthritis (RA) can be treated or prevented (Li et al., 2020). In addition, TNF-α can activate caspase-8/3, ultimately stimulating apoptosis, which may also lead to skeletal muscle damage (Wu et al., 2023). Cell experiments further verified the effect of Shenqi Fuzheng Injection on alleviating mitochondrial damage in myoblasts and reducing apoptosis.

We emphasise that molecular docking provides a measure of predicted binding affinity, not functional agonism or antagonism, and that the calculated poses use isolated compounds at arbitrary concentrations rather than the actual circulating concentrations achieved after intravenous SFI administration. Because SFI is a multi-component preparation, the biological relevance of the docking predictions is anchored by the whole-formula cell experiments, which showed that SFI at clinically scaled concentrations (5–20 mg/mL crude-drug equivalent) attenuates FaDu-induced C2C12 apoptosis and mitochondrial membrane-potential loss. Future pharmacokinetic studies using LC-MS/MS are needed to quantify the exact plasma levels of quercetin, kaempferol and luteolin after SFI infusion.

Through network pharmacology, the key components Quercetin, Kaempferol, and Luteolin in SFI were identified as having strong binding affinity with Caspase-3, Caspase-8, TNF, MAPK3, and TP53, with cell experiments further validating the anti-apoptotic and mitochondria-protective effects.

Regarding study scope: The FaDu/C2C12 co-culture model employed in this study is recognized to better recapitulate cancer-associated muscle wasting than purely age-related sarcopenia. Since sarcopenia is highly prevalent in cancer patients (estimated 15%–57% depending on cancer type and diagnostic criteria), and since cancer-associated muscle wasting and age-related sarcopenia share key molecular pathways (TNF-α/Caspase cascade, mitochondrial dysfunction, apoptotic signaling), we positioned this study as addressing both contexts. The identified molecular targets (Caspase-3/8, TNF, TP53, MAPK3) are well-established across multiple etiologies of skeletal muscle atrophy.

Regarding database limitations: The current study used TCMSP, TCMID, and CTD as primary databases. We acknowledge that future studies should incorporate SwissTargetPrediction for structure-based compound target prediction, and GeneCards, OMIM, and PharmGKB for more comprehensive disease target retrieval, which would enrich the identified target network.

Regarding the undifferentiated cell model: This study employed undifferentiated C2C12 myoblasts rather than fully differentiated myotubes. Since sarcopenia primarily involves dysfunction of mature muscle fibers, future studies should validate these findings using differentiated myotubes, primary human myosatellite cells, or *in vivo* animal models to better recapitulate the sarcopenic phenotype.

Existing clinical evidence for SFI: SFI has been evaluated in numerous clinical trials as an adjuvant therapy in cancer patients, demonstrating improvements in cancer-related fatigue, immune function, and quality of life—outcomes directly relevant to cancer-associated muscle wasting. These published clinical observations provide contextual support for the therapeutic relevance of SFI in the cancer-muscle wasting axis, although prospective clinical studies specifically targeting muscle mass and function are needed. The biological validity of the present network pharmacology investigation is further supported by converging pre-existing evidence: (1) the key SFI components identified herein—quercetin, kaempferol, and luteolin—have been independently shown to attenuate skeletal muscle atrophy, reduce inflammatory cytokine levels (TNF-α, IL-6), inhibit Caspase-mediated apoptosis, and preserve mitochondrial function in preclinical models (Koshinaka et al., 2020; Eid et al., 2010; Tang, 2016; Le et al., 2014; Qu et al., 2021; Böttcher et al., 2025; Kim et al., 2023; Kodaka et al., 2020); (2) the core targets identified (Caspase-3/8, TNF, TP53, MAPK3) are well-established molecular nodes in the pathophysiology of cancer-associated muscle wasting across multiple preclinical and clinical studies; (3) our cell experiments directly validate Caspase-3/8 inhibition and mitochondrial protection by SFI in a cancer-muscle co-culture system, providing direct experimental evidence as a biological anchor for the network pharmacology predictions. These converging lines of evidence constitute the requisite biological foundation supporting the scientific rationale of this exploratory investigation.

## 5 Limitations

This study has several important limitations that should be explicitly acknowledged: (1) Inapplicability of OB/DL criteria to injectable formulations: The OB ≥ 30% and DL ≥ 0.18 criteria are pharmacokinetic parameters designed for oral drugs and are physiologically inapplicable to SFI, which is administered intravenously. This represents an inherent bias in compound screening that is shared by most published network pharmacology studies on injectable TCM preparations due to current database limitations. Future studies should employ LC-MS/MS-based profiling to directly identify the active components in SFI. (2) Absence of *in vivo* pharmacodynamic data in the present manuscript: No animal experiments or clinical studies are included here to confirm the therapeutic efficacy of SFI in sarcopenia. An independent *in vivo* study using tumour-bearing cachexia models with measurements of gastrocnemius mass, grip strength, muscle fibre cross-sectional area, and candidate target expression is currently underway; those data will be reported separately and cannot be incorporated into this manuscript (Fearon et al., 2011; Argilés et al., 2014). All mechanisms proposed here remain hypothesis-generating until validated *in vivo*. (3) Undifferentiated cell model: C2C12 myoblasts rather than differentiated myotubes were used, limiting direct applicability to mature muscle-fibre biology. Consequently, markers of myotube maturation (myotube diameter, fusion index, myosin heavy-chain expression) and protein-degradation markers (Atrogin-1/MuRF1) were not assessed in this exploratory screen; validation in differentiated myotubes, primary human myosatellite cells or *in vivo* cachexia models is planned in the ongoing follow-up study. (4) Database selection: The use of TCMSP/TCMID/CTD without supplementary databases (SwissTargetPrediction, GeneCards, OMIM) may result in incomplete target coverage.

## 6 Conclusion

Based on the TCM theory of ‘spleen governing muscles’, Shenqi Fuzheng Injection exhibits a complex mechanism of action in the treatment of sarcopenia. This exploratory study demonstrates that key active components (Quercetin, Kaempferol, Luteolin) in SFI have strong binding affinity with core targets including Caspase-3, Caspase-8, MAPK3, TNF, and TP53, and that SFI can effectively alleviate mitochondrial damage and inhibit apoptosis in myoblasts in a cancer-associated co-culture model. These findings provide a preliminary computational and cellular framework for understanding SFI’s potential mechanisms. *In vivo* animal experiments and clinical studies are required to confirm these predictions and validate the therapeutic efficacy of SFI in cancer-associated muscle wasting.

## Data Availability

The original contributions presented in the study are included in the article/supplementary material, further inquiries can be directed to the corresponding author.
